# A Composite Model for Subgroup Identification and Prediction via Bicluster Analysis

**DOI:** 10.1371/journal.pone.0111318

**Published:** 2014-10-27

**Authors:** Hung-Chia Chen, Wen Zou, Tzu-Pin Lu, James J. Chen

**Affiliations:** 1 Division of Bioinformatics and Biostatistics, National Center for Toxicological Research, U.S. Food and Drug Administration, Jefferson, Arkansas, United States of America; 2 Graduate Institute of Biostatistics and Biostatistics Center, China Medical University, Taichung, Taiwan; 3 Department of Public Health, Graduate Institute of Epidemiology and Preventive Medicine, National Taiwan University, Taipei, Taiwan; Semmelweis University, Hungary

## Abstract

**Background:**

A major challenges in the analysis of large and complex biomedical data is to develop an approach for 1) identifying distinct subgroups in the sampled populations, 2) characterizing their relationships among subgroups, and 3) developing a prediction model to classify subgroup memberships of new samples by finding a set of predictors. Each subgroup can represent different pathogen serotypes of microorganisms, different tumor subtypes in cancer patients, or different genetic makeups of patients related to treatment response.

**Methods:**

This paper proposes a composite model for subgroup identification and prediction using biclusters. A biclustering technique is first used to identify a set of biclusters from the sampled data. For each bicluster, a subgroup-specific binary classifier is built to determine if a particular sample is either inside or outside the bicluster. A composite model, which consists of all binary classifiers, is constructed to classify samples into several disjoint subgroups. The proposed composite model neither depends on any specific biclustering algorithm or patterns of biclusters, nor on any classification algorithms.

**Results:**

The composite model was shown to have an overall accuracy of 97.4% for a synthetic dataset consisting of four subgroups. The model was applied to two datasets where the sample’s subgroup memberships were known. The procedure showed 83.7% accuracy in discriminating lung cancer adenocarcinoma and squamous carcinoma subtypes, and was able to identify 5 serotypes and several subtypes with about 94% accuracy in a pathogen dataset.

**Conclusion:**

The composite model presents a novel approach to developing a biclustering-based classification model from unlabeled sampled data. The proposed approach combines unsupervised biclustering and supervised classification techniques to classify samples into disjoint subgroups based on their associated attributes, such as genotypic factors, phenotypic outcomes, efficacy/safety measures, or responses to treatments. The procedure is useful for identification of unknown species or new biomarkers for targeted therapy.

## Introduction

Recent advances in biotechnology have generated great interest in the development of statistical methods and data mining techniques to analyze massive amounts of biological and medical data for understanding biological processes, discovering new species, or identifying new biomarkers for safety assessment, disease diagnostics and prognostics, and prediction of treatment response, etc. For example, metagenomics utilizes DNA sequence data to detect and identify representative species in environmental and clinically relevant samples and to discover genes or organisms with novel or useful functional properties [1]–[4].

In clinical treatment, patients are heterogeneous due to differences in genetic pre-dispositions, lifestyle, and disease characteristics. Personalized medicine utilizes genomic predictors of target patient population for assignment of more effective therapies to ensure safety and avoid adverse events or unnecessary treatment [5], [6]. A main goal is to develop a procedure that can classify patients into subgroups representing different disease characteristics or different responses to a specific treatment. For example, acute lymphoblastic leukemia (ALL) is a heterogeneous disease, including several subtypes (T-ALL, E2A-PBX1, BCR-ABL, TEL-AML1, MLL) differing in their response to chemotherapy [7]–[9]. Identifying important leukemia subtypes to accurately assign patients to specific risk/treatment groups is a difficult and expensive process, requiring the combined expertise of hematologist/oncologist, pathologist, and cytogeneticist [9].

In food safety surveillance, serotyping of pathogen strains is usually the first important step for identification and characterization of *Salmonella* isolates in outbreak investigations. However, standard methods for serotype identification of strains are tedious and time-consuming [10], [11]. Considering there are over 2,500 outbreak strains of unknown or new serotypes, development of a procedure for early and fast screening and source tracking is essential. PFGE (pulsed-field gel electrophoresis) genotyping method has been used to investigate the relatedness of individual cases, and to confirm an outbreak of a disease and determine its possible source [10]–[13]. Previous works [10], [11], [14]–[16] reported that serotypes of *Salmonella* isolates could be deduced and predicted based on PFGE fingerprints. Thus, PFGE fingerprint profiling using data mining algorithms can potentially provide a possible alternative method for fast screening and identifying *Salmonella* serotypes.

In the aforementioned applications, the primary goal is to develop a class prediction model that can accurately identify population subgroups (cancer or strain subtypes) for new samples. There are three main aims: 1) classifying samples into distinct subgroups from large and complex unlabeled multivariate data, 2) characterizing the relationships among the subgroups identified, and 3) developing a prediction model to classify subgroup memberships of new samples by finding a set of predictor variables.

Classification is the standard approach to developing a model for class prediction of new samples. Classification is a supervised analysis, in which each sample has a predefined class label. A classification model builds a mathematical function for predicting class memberships of new unlabeled samples by learning the relationships between the class memberships of samples and their attributes from the sampled data [17]–[21]. The objective of this learning is to search for a prediction function and a least number of predictor variable that maximizes the probability of classification accuracy. In other words, a classification model utilizes class label information to optimize predictive accuracy. Without class labels, classification analysis is not viable for sample classification and prediction. Furthermore, standard classification algorithms are only applicable to the samples from the classes that are present in the sampled data. The algorithms are incapable of classifying the samples from classes other than those presented within the dataset, such as classifying new cancer subtypes in clinical medicine or new serotypes in pathogen identification.

Cluster analysis is the standard data mining technique for identification of structures and patterns in the data by partitioning samples into disjoint subgroups and finding their relationships. There are hierarchical and non-hierarchical clustering algorithms. The hierarchical algorithm clusters the objects into a tree-like dendrogram [22]. The hierarchical clustering method can provide the relationship among the samples or the clusters; however, it is inefficient for determining subgroups when the number of samples is large. The non-hierarchical clustering algorithms divide objects into a pre-specified number of groups; k-means [23] and self-organizing maps (SOM) [24] are two commonly known algorithms. Specification of the number of subgroups is a challenge when the number of subgroups is large.

Clustering techniques provide a global analysis of samples by partitioning samples with similar attributes in the same cluster. Each sample is assigned to one and only one cluster, based on all attributes. In many applications, such as gene expression experiments, functionally related genes may exhibit a similar pattern only in a subset of patients with certain medical conditions, not in all patients; also, some genes may involve more than one function or no function at all, and associate with more than one condition or no condition. A primary goal in these applications is to identify those subsets of co-expressed/co-regulated genes with associated subsets of samples with similar conditions. Cluster analysis cannot effectively identify the substructures between a subset of genes and a subset of samples. Biclustering analysis provides an approach to identify substructures in the sampled data. Biclustering techniques identify biclusters by simultaneously clustering both samples and attributes [25]–[39]. Each bicluster is defined as a subset of attributes associated with a subset of samples. For an overview of biclustering methods see the reviews of Madeira and Oliveira [28] and Kriegel et al. [32]. Alternatively, Baker el al. [40], [41] developed GeneWeaver system aiming to integrate multiple data sources to identify associations between phenotypes and gene sets. The system was capable of demonstrating the clustered genes and phenotypes as hierarchical associations. Recently, Zhang et al. [42] further developed an approach to finding maximum bicliques in bipartite graphs, which was incorporated into the GeneWeaver system. Bicluster analysis can be viewed as an application of GeneWeaver to identify substructures in single study.

Both cluster and bicluster analyses are unsupervised analyses, in which samples do not have a predefined class label. These two methods are effective techniques for subgroup identification and characterization, but, are inefficient for subgroup prediction. Several supervised biclustering procedures have been proposed for classification of labelled sample datasets [43]–[46]; these methods incorporate label information into the process of building biclusters. More discussion in the use of cluster/bicluster analysis for prediction and supervised biclustering procedures are given in the Discussion section.

In this paper, we propose a composite modeling approach for subgroup identification and prediction via a bicluster analysis. The proposed approach combines an unsupervised biclustering technique to identify potential sample subgroups in the first step, and a supervised classification technique to predict sample subgroup memberships in the second step. The proposed composite model neither depends on any specific biclustering algorithm or patterns of biclusters, nor on any classification algorithms. Any biclustering methods can be used in the first step of bicluster identification. This paper uses a SVD-based biclustering algorithm to identify constant biclusters [39]; this method has been shown to perform well in extensive comparisons with various biclustering methods, and found to be generally superior in terms of sensitivity and specificity. The primary focus of this paper is subgroup classification and prediction. Three well-known classification algorithms are considered in the second step of subgroup classification and prediction: support vector machine [17], [18], random forests[19], and diagonal linear discriminant analysis [21]. The proposed composite model for subgroup identification and prediction is applied to a synthetic dataset and three real datasets for illustration.

## Methods

Consider a two-way data matrix with rows representing the measured attributes and columns representing samples. Many singular value decomposition (SVD) approaches for bicluster analysis of microarray data have been proposed and demonstrated to be effective [34]–[39]. In this paper, a SVD-based biclustering method [39] was used to identify substructures between subsets of attributes and subsets of samples. An advantage of SVD-based biclustering methods is that the biclustering results do not depend on the random starting seeds. In the proposed approach, first a set of biclusters was identified using the SVD-based biclustering method [39], followed by generating a set of binary classifiers, each built from one of the biclusters identified. A composite model is then developed to classify samples into disjoint subgroups described below.

**Figure 1:**
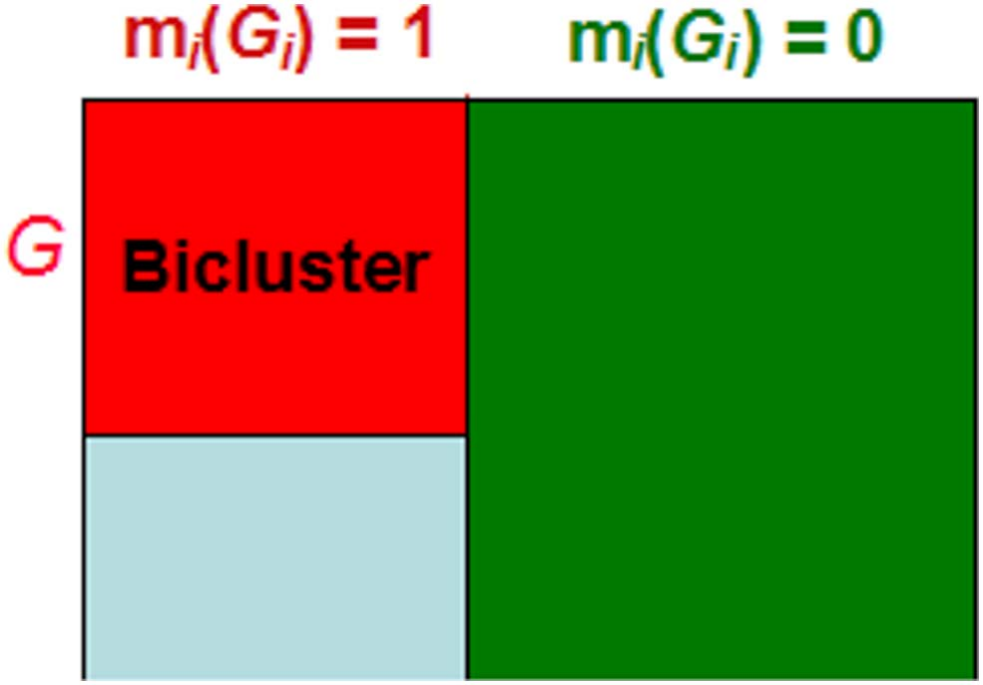
Subgroup-specific binary classifier. For each bicluster C*_i_* = {G*_i_*, S*_i_*}, a subpopulation-specific genomic binary classifier m*_i_*(G*_i_*) = *I*{s ∈ *S_i_*}, is built to predict where or not a sample is in the subpopulation S*_i_*, where I is an indicator function.

Denote the collection of biclusters identified as C = {C*_1_*, C*_2_*,…,C*_k_*}. Each bicluster C*_i_* consists of a subset of samples S_i_ that have similar attributes G*_i_* (*i* = *1,.,k*). Thus, each S*_i_* represents a subgroup in the sampled population. A subgroup-specific binary classifier m*_i_* can be built to determine whether or not a sample *s* with the attribute ***g*** is in the associated subgroup S*_i_*, that is, m*_i_*(***g*** *|G_i_*) = *I*{s ∈ *S_i_*}, where I is an indicator function (Figure 1). A composite classification model M, which consists of the collection of the binary classifiers M = {m*_1_*, …, m*_k_*}, is developed to partition samples into several disjoint subgroups described below.

For a given sample *s* with the attribute ***g*** *,* each component binary classifier predicts whether or not the sample *s* belongs to its corresponding subgroup, where there are *k* predictive outcomes. Denote yes as “1” and no as “0”. Suppose the composite classification model consists of five binary classifiers (m*_1_*,…,m*_5_*) with the corresponding subgroups (S*_1_*, …, S*_5_*). For example, the outcome (1,0,0,0,0) of the composite model implies that the sample is in S_1_, (0,0,1,1,0) implies that the sample is in S*_3_* and S*_4_*, and (0,0,0,0,0) implies that the sample is not in any of the five subgroups. For *k* binary classifiers, there are 2*^k^* possible patterns of predictive outcomes. Each pattern represents a subgroup. However, when *k* is modest or large, many patterns would contain very few samples or no samples at all. When the number of patterns is large, a minimum of n* = 5–10 samples may be set as the criterion to form a (major) subgroup for further analysis. The patterns that contain less than n* samples are referred to as minority subgroups.

Binary classifiers can be developed using any classification algorithms. This paper uses the three well-known algorithms: support vector machine (SVM) [17], [18], random forests (RF) [19], and diagonal linear discriminant analysis (DLDA) [21]. These three algorithms were shown to perform well and have been the most popular classification algorithms for class prediction of high dimensional data [47].

In the development of a classification model, the most important consideration is to unbiasedly evaluate its “performance”. The common measures of performance are sensitivity (the proportion of correct positive classifications out of the number of true positives), specificity (the proportion of correct negative classifications out of the number of true negatives), and accuracy (the total number of correct classifications out of the total number of samples). Procedures with both high sensitivity and high specificity will have high accuracy. To obtain unbiased estimates, the current sampled data are divided into a training set and a separate test set [48]; the training set is used for model development, and the test set is used for performance assessment. The split-sample and cross-validation methods are commonly used to evaluate performance of a classifier. The split-sample method randomly splits the data into two subsets from either the entire data or a designated test dataset. Split-sample validation is useful when the sample size is large. Cross validation involves repeatedly splitting the sampled data into a training set and test set to generate different training and test sample partitions to repeatedly estimate “accuracy” measures. Leave-one-out is a cross validation in which one sample is left out as a test set while all the other samples constitute the train set. The “accuracy” measures are estimated after all samples are tested. This paper uses both leave-one-out and split-sample for performance evaluation.

## Results

### Simulation Experiment

**Figure 2:**
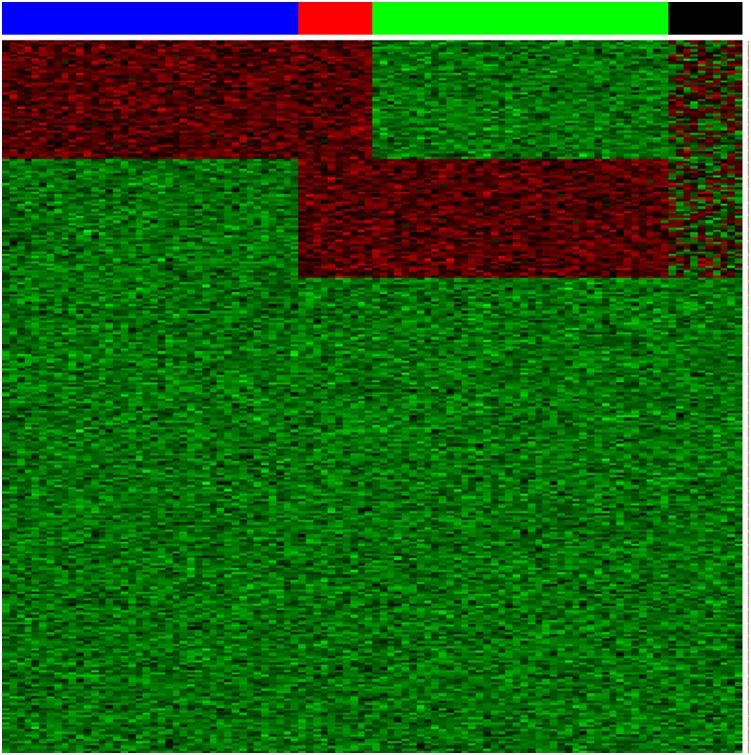
A synthetic 300×100 data matrix consists of two main bicluster regions with the size of 50×50 having 10 overlapping columns. The columns represent 100 samples consisting of 4 subgroups: S1 (columns 1–40, blue), S2 (columns 41–50, red), S3 (columns 51–90, green), and S4 (columns 91–100, black); the first 100 rows represent attributes: G1 (rows 1–50), G2 (rows 1–100), G3 (rows 51–100), and G4 (rows 1–100).

A simulation experiment was conducted to illustrate the proposed approach using asynthetic dataset of size 300 (rows)×100 (columns). The dataset consisted of two main bicluster regions with the size of 50×50 having 10 overlapping columns. The first main bicluster consisted of rows 1–50 and columns 1–50, and the second bicluster consisted of rows 51–100 and columns 41–90. The remaining columns 91–100 were in neither biclusters. The bicluster (signal) data were generated from the normal distribution N (11,1.22) and background data were generated from the normal random variable N (6, 1). For masking purpose, random signals were also generated in the first 100 attributes for the last 10 samples. This dataset can be summarized as four biclusters as follows. The columns represent 100 samples consisting of 4 subgroups: S1 (columns 1–40, blue), S2 (columns 41–50, red), S3 (columns 51–90, green), and S4 (columns 91–100, black); the first 100 rows represent attributes: G1 (rows 1–50), G2 (rows 1–100), G3 (rows 51–100), and G4 (rows 1–100) (Figure 2).

Applying the SVD-based biclustering method [39] to the permutated dataset, four bicluster regions were identified. The dimensions of the four biclusters, C*_1_*, C*_2_*, C*_3_*, and C*_4_* were 100×16, 50×51, 50×58, and 100×15, respectively. Three classification algorithms were then used to develop four binary classifiers m*_1_*, m*_2_*, m*_3_*, and m*_4_*. There were 16 possible patterns.

Table 1 (upper panel) lists those 6 patterns with their frequencies from the SVM algorithm, where the column labels the true sample subgroup. Among the 16 possible patterns, there were major subgroups (n≥5) and three minor subgroups (n<5), and the remaining 10 patterns have no samples. Three major subgroups were (0,0,1,0), (0,1,0,0), and (1,1,1,1) identifying *S_1_*, *S_3_*, and *S_2_*, respectively. The sensitivity and specificity are shown in the last two rows. The overall accuracy is 0.90. All the subgroups *S_1_–S*_3_ were identified correctly. A test dataset consisting of 1,000 samples were generated for performance evaluation. The four subgroups were generated according to the probabilities 0.4, 0.1, 0.4, and 0.1 in contrast to the training set where the numbers of four subgroups were fixed at 40, 10, 40, and 10. The sensitivity and specificity for the 1,000 simulated samples were calculated for each of the four subgroups. The procedure was repeated 1,000 times. The averaged sensitivity and specificity over the 1,000 repetitions were shown in Table 1 (lower panel). The averaged accuracy is 0.974. The sensitivity was 0.654 for S4; since the samples sample size was 1,000, the number of S4 samples was about 100 in each evaluation. Unlike the analysis of training samples, sufficient number of data from S4 was generated to form a subgroup and identified.

**Table 1:** Upper panel, frequency distributions of classification patterns identified by the SVM composite model (m1, m2, m3, m4) for the synthetic training dataset consisting of 4 subgroups, S1, S2, S3, and S4; Lower panel, performance of the SVM composite prediction model for the test dataset of 1,000 simulated samples.

SubgroupPattern	S1(n = 40)	S2(n = 10)	S3(n = 40)	S4(n = 10)	Total(n = 100)
**Training**					
0010	**40**	0	0	4	44
0100	0	0	**40**	2	42
1111	0	**10**	0	1	11
0110	0	0	0	1	1
0111	0	0	0	1	1
1011	0	0	0	1	1
Sensitivity	1	1	1	0	0.90
Specificity	0.93	0.99	0.97	1	0.98
**Test**					
Sensitivity	1.000	0.994	1.000	0.654	0.964
Specificity	0.968	0.997	0.963	0.999	0.990

Table values are the averages over 1,000 repetitions.

Tables S1 and S2 are the results from the RF and DLDA algorithms, respectively. The performances of the three algorithms are similar, in general. All three algorithms show high sensitivity and specificity in identifying (test) the *S_1_* and *S_3_* samples. *S_2_* has the attributes across two subgroups *S_1_* and *S_3_*. *S_4_* was designed to have indefinite attributes and difficult to be identified. The pattern corresponding to *S_2_* is (1,1,1,1) using SVM and DLDA, and the pattern is (1,1,0,0) using RF. SVM appears to perform slightly better than RF and DLDA. For *S_4_*, as expected, the sensitivity is low in all three algorithms.

### Analysis of a lung cancer dataset

A public lung cancer microarray dataset was used to evaluate the performance of the proposed procedure and compare with k-means cluster analysis. The dataset was from a study (GSE3141) of using gene expression signatures to identify patterns of oncogenic pathway deregulation in lung cancer subtypes [49]. The original GSE3141 dataset was retrieved from the Gene Expression Omnibus [50]. The dataset consisted of 111 lung cancer samples with 53 adenocarcinoma (AD) and 58 squamous cell carcinoma (SQ) subtypes. This analysis was performed to distinguish these two lung cancer subtypes assuming no information on the sample subtypes. In the analysis, a quantile normalization algorithm was performed to remove the systematic biases. For each probe, standard error was calculated across all samples and ranked decreasingly. The top 100 probes with the largest standard errors were selected as attribute variables.

**Figure 3:**
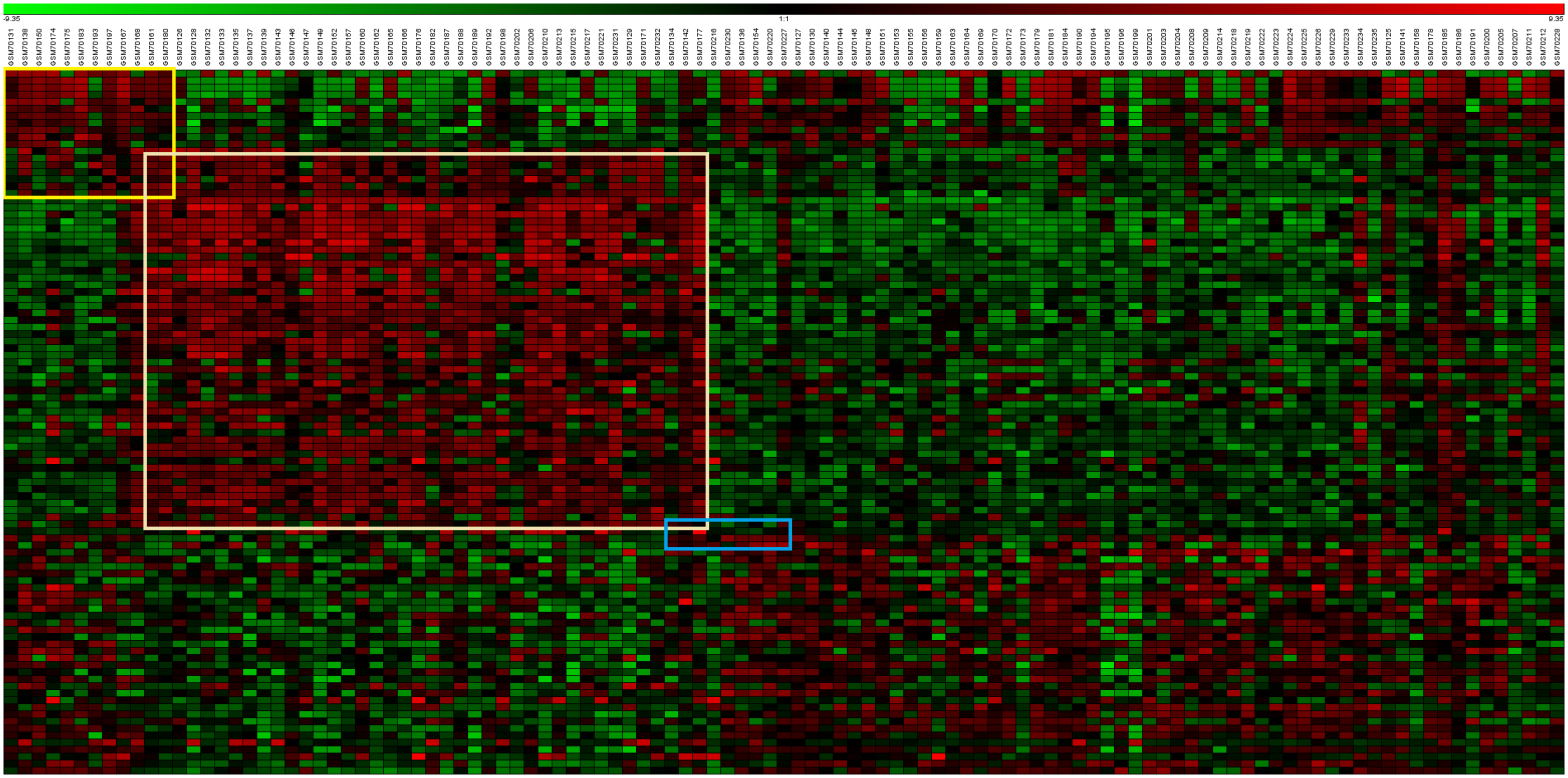
Lung Cancer data: three biclusters are identified, 55×40, 18×22 and 4×10, using top 100 genes.

The proposed approach was performed on the data matrix of 100 genes by 111 samples. The bicluster analysis identified 32 biclusters. A cutoff of at least 10 samples was used to eliminate small biclusters, such as sizes of 2×2 or 2×3, resulting in 3 clusters (Figure 3). The sizes of the three biclusters were 55×40, 18×22, and 4×10. A composite model M = {m*_1_*, m*_2_*, m*_3_*} was built based on the three biclusters. The leave-one-out cross (LOU) validation was used to classify each sample into one of the possible 8 subgroups.

Table 2 shows the results from the composite models and k-means methods for k = 2, 3, 4. Note that unlike the composite model using LOU, all 111 samples were used in the k-means analysis. The SVM algorithm identified three patterns (0,0,0), (0,1,0), and (1,0,0), while RF and DLDA identified four patterns (0,0,0), (0,1,0), (1,0,0), and (1,1,0). The classifier m_1_ generated from the bicluster C*_1_* appears to be associated with the SQ subtype. Note that the classifier m*_3_* by itself or in combination with m*_1_* and m*_2_* assigned none samples in a subgroup. That is, all samples, including 10 samples from C_3_ were not in C_3_, as predicted by m_3_. Based on the majority rule, SVM, RF, and DLDA correctly identified 41, 40, and 42 out of the 52 AD subtypes, respectively. All three algorithms identified 52 out of the 58 SQ subtypes. The performance between the composite models and 2-mean are generally similar. The 52 SQ subtypes identified by the 2-means and by the three composite models are identical. The 42 AD subtypes identified by 2-means contained those 41, 40, and all 42 ADs identified by the SVM, RF, and DLDA composite models, respectively. Using n* = 5, SVM identified two subgroups, including 39 AD and 52 SQ subtypes; RF identified two subgroups of 38 AD and 50 SQ subtypes; DLDA identified four subgroups with 42 AD and 52 SQ subtypes.

**Table 2:** Subgroup classification for the 111 lung cancer patients of the GSE3141 dataset using the composite model with the SVM, RF and LDA algorithms, and K-means (2-means, 3-means and 4-means) cluster analysis.

Methods	Subgrouppattern	Adenocarcinoma	Squamouscellcarcinoma
SVM	000	39	6
	010	2	0
	100	12	52
RF	000	38	5
	010	2	1
	100	13	50
	110	0	2
DLDA	000	35	4
	010	7	2
	100	11	37
	110	0	15
2-Means	0	42	6
	1	11	52
3-Means	0	32	3
	1	14	3
	2	7	52
4-Means	0	33	4
	1	9	2
	2	6	22
	3	5	30
	Total	53	58

In the 4-means analysis, Groups 0 and 1 were from the split of Group 0 in the 2-means analysis, and Groups 2 and 3 were from the split of Group 1. However, the results of the 3-means analysis were peculiar. For example, there were 32, 14, and 7 adenocarcinomas for Groups 0, 1, and 2, respectively. Comparing with the 4-means analysis, the 32 consisted 21, 9, and 2 from Groups 0, 1, and 2, respectively; similarly, the 14 consisted of 12 and 3 from Groups 0 and 2, respectively.

### Analysis of the breast cancer dataset

The dataset of van’t Veer et al. [51] contained 97 breast cancers (46 from patients who developed distant metastases within 5 years and 51 from patients who continued to be disease-free after a period of at least 5 years). The outcome was cancer-related survival time with 6391 genes as predictor variables.

**Figure 4:**
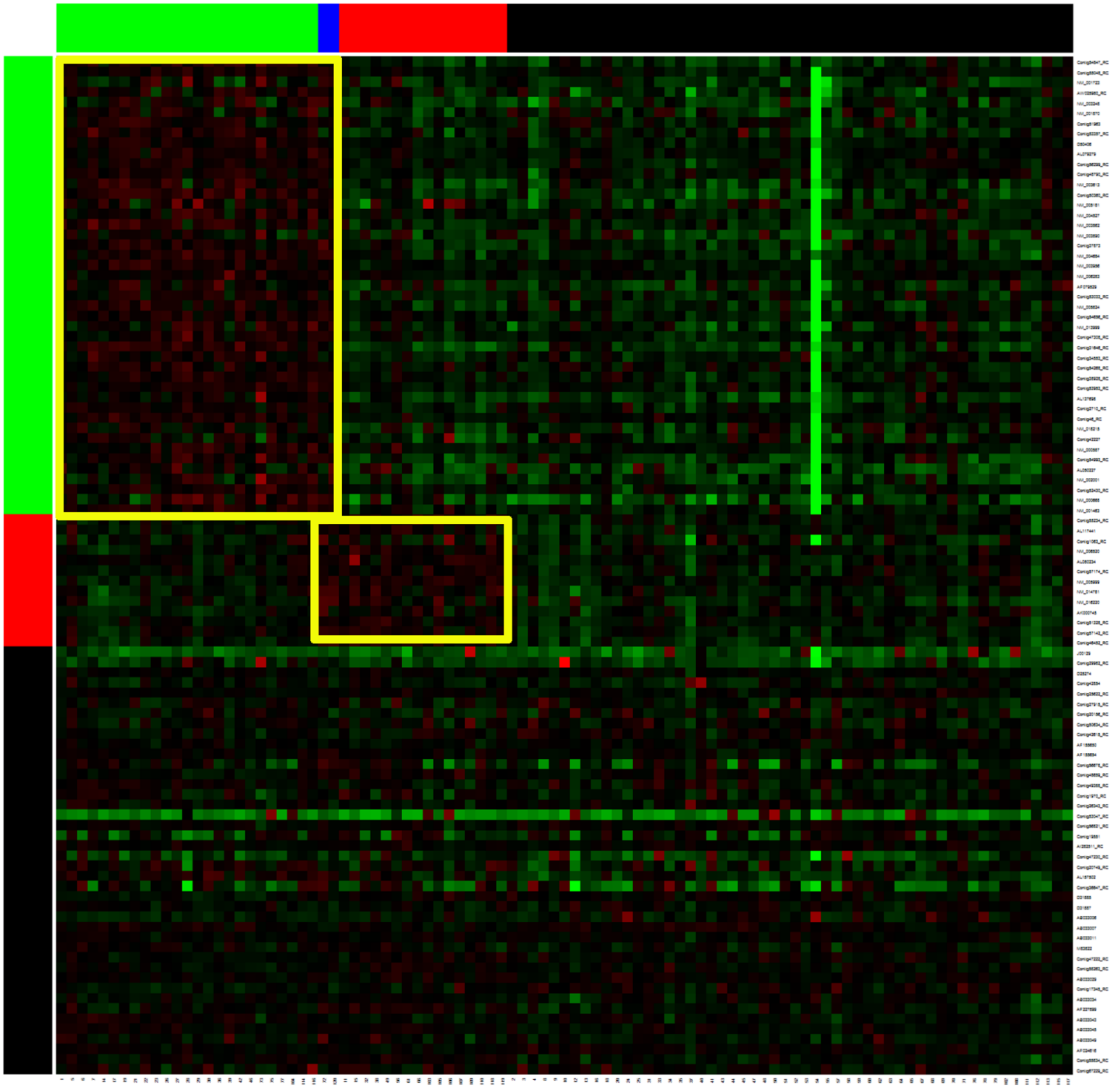
Breast Cancer data: two biclusters are identified, 45×27 and 13×18, using 6391 genes 100 of which are demonstrated.

**Figure 5:**
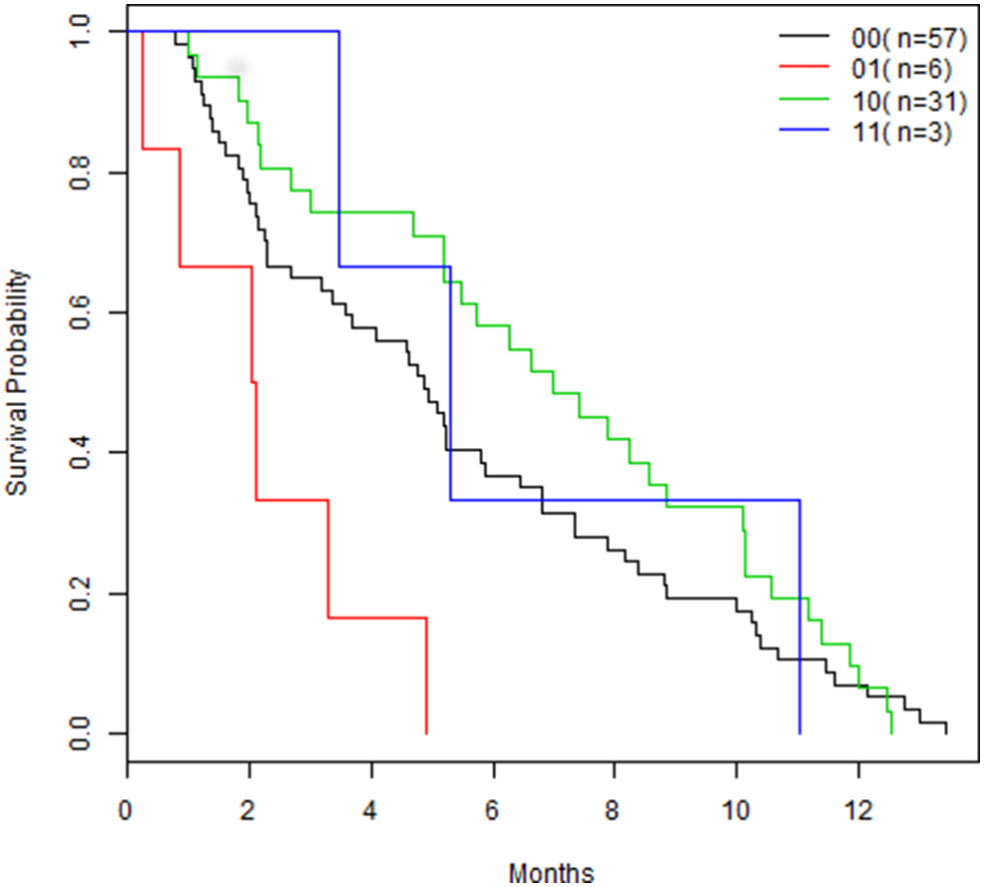
The prediction model divided the 97 patients into four subgroups using SVM. The logrank test for differences among the four subgroups (0,0), (0,1), (1,0), and (1,1) was 0.003.

Two biclusters with dimensions of 45×27 and 13×18 were identified from the 6391 genes and 97 patients (Figure 4). Two patients belonged to both biclusters; two binary classifiers, m_1_ and m_2_, were developed. The leave-on-out cross validation analysis divided the 97 patient into 4 subgroups. Table 3 shows the results from the composite models. The m_1_ classifier identified low risk group patients and m_2_ identified high risk group patients. Figure 5 shows the plots of the survival time for four subgroups from the SVM model. Figures S1 and S2 are the plots from the RF and DLDA composite models, respectively. The logrank tests for the differences between the two major subgroups (0,1) versus (1,0) were 0.284, 0.510, and 0.599 for SVM, RF, and DLDA, respectively.

**Table 3:** Subgroup classification for the 97 breast cancer patients (46 from patients who developed distant metastases within 5 years and 51 from patients who continued to be disease-free after a period of at least 5 years) using the SVM, RF, and DLDA composite models.

Subgroup pattern	SVM	Random Forest	DLDA
(0,0)	57	64	47
(0,1)	6	4	15
(1,0)	31	27	28
(1,1)	3	2	7
Logrank test for (0,1) vs (1,0) subgroups	0.284	0.519	0.599

### Analysis of the Salmonella isolate dataset

The *Salmonella* isolate dataset consisted of 45,924 PFGE isolates covering 32 mostly encountered serotypes published by Zou et al. [16]. The sample isolates were genotyped by the Pulsed-Field Gel Electrophoresis (PFGE) with DNA bands representing the presence and absence of a feature in a location as a fingerprint of isolates. Each isolate has 60 or 61 bands. Five serotypes, I4,[5],12:i-, Hadar, Oranienburg, Thompson, and Typhimurium, were randomly selected for data analysis. Each serotype consisted of about 2,000 isolates. The analysis was to illustrate the use of the proposed composite model to identify the five serotypes and their subtypes, if any, and evaluate its performance as compared with the k-means clustering and SVM and RF classifications when the test set contained isolates from the serotypes that are not observed in the training set. The DLDA algorithm was not considered in this example since the PFGE fingerprints were binary features.

The data were first randomly divided into a training and a test dataset for each serotype. The bicluster analysis identified 10 biclusters and built 10 binary classifiers (m_1_–m_10_) from the training dataset. The SVM and RF composite models then were applied to each training sample; 16 patterns were identified. The SVM model identified 16 patterns. Based on n* = 5 as a cutoff, 13 subgroups were identified (Table 4). Note that the classifier m*_9_* and m*_10_* by itself or in combination with other classifiers assigned no samples into a subgroup. The interpretation (and presentation) of the performance was based on the known serotypes; in the analysis, the serotype was determined by majority rule. The sensitivities were from 86% to 99%, specificities were high at least 99%, except the Typhimurium with 93.2%. The overall accuracy was 94.2% and specificity was 98.2%.

**Table 4:** Frequency distributions of classification patterns identified by the SVM composite model (m_1_–m_10_) for the *Salmonella* PFGE training dataset consisting of five serotypes.

13Subgroups(n≥5)	4,5,12:i-n = 1113	Hadar n = 982	Oranienburgn = 997	Thompsonn = 990	Typhin = 972	Totaln = 5054
0000000000	27	73	126	49	142	417
1000000000	653	0	0	0	0	653
1000000100	211	0	0	0	0	211
1000001000	212	0	0	0	0	212
0000001000	6	0	0	0	0	6
0100000000	0	0	0	0	829	829
0010000000	0	1	0	940	0	941
0001000000	0	0	34	0	0	34
0000100000	0	0	215	0	0	215
0001100000	0	0	593	0	0	593
0001110000	0	0	10	0	0	10
0000110000	0	0	9	0	0	9
0000010000	0	905	10	1	1	917
0000000100	2	0	0	0	0	2
0000011000	2	0	0	0	0	2
0010010000	0	3	0	0	0	3
Correctidentification	1082	905	861	940	971	4759
Sensitivity	0.967	0.922	0.864	0.950	0.999	0.942
Specificity	1	0.997	1	1.000	0.932	0.982

Sixteen classification patterns are identified; 13 of the 16 have frequencies of at least 5 (last column). The last two rows show the sensitivity and specificity of the model performance.

The RF model identified 15 patterns, one less than the SVM model (Table S3). The difference is in the serotypes 4,5,12:i- identification. In the SVM classification, there were two patterns (1000000000) and (1000000100) with 653 and 211 for a total of 864 isolates, respectively. In the RF classification, there was no (1000000100) pattern, instead, the pattern (1000000000) consisted of 898 isolates. In addition to m*_9_* and m*_10_*, m_8_ assigned no samples into a subgroup. Based on n* = 5 as a cutoff, 12 subgroups were identified. The sensitivities were from 86% to 99%, specificities were 93% to 100%. The overall sensitivity (accuracy) was 94.2% and specificity was 98.2%.

The SVM and RF composite models were applied to the test dataset, which included 1,000 additional samples (named “Decoy”) from the serotypes other than the five training serotypes. The analysis of the test dataset classification described below is for the SVM composite model, the results for the RF composite model are given in Table S4.

**Figure 6:**
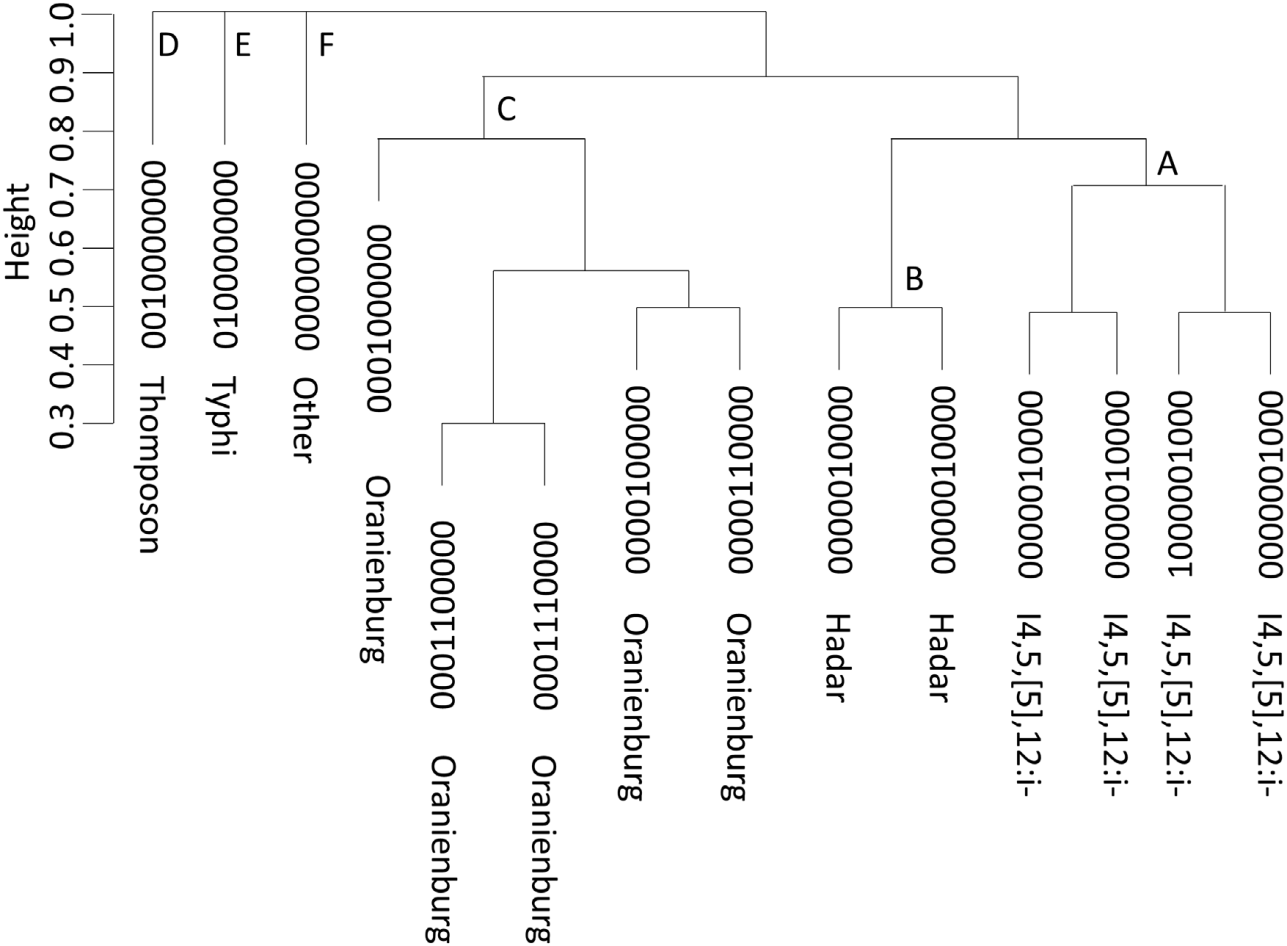
Hierarchical cluster analysis of the 14 subgroups identified from the test dataset using the average linkage distance. The 14 subgroups consist of 5 major subgroups: 1. Thompson (0010000000); 2. Typhimurium (0100000000); 3. Decoy (0000000000); 4. Oranienburg (0001000000, 0000100000, 0001100000, 0001110000, 0000110000); 5. Hadar (0000010000, 1000010000) and I4,[5],12:i- (1000000000, 1000000100, 1000001000, 0000001000).

The SVM model identified 24 classification patterns from the 10 binary classifiers m_1_–m_10_. Based on the n* = 5 as a cutoff, 14 subgroups were identified (Table 5), where 13 of the 14 were identical to the 13 subgroups that were identified in the training data. The additional subgroup consisted of 8 Hadar isolates. The serotypes and their associated binary classifiers were: 4,5,12:i-: m_1_, m_7,_ (m_1,_ m_7_)_,_ (m_1,_ m_8_); Hadar: m_6,_ (m_1_, m_6_); Oranienburg: m_4_, m_5,_ (m_4_, m_5_)_,_ (m_5_, m_6_)_,_ (m_4_, m_5_, m_6_); Thompson: m_3_; Typhimurium: m_2_.The sensitivities between the training and test datasets were similar for the data of the five training serotypes. The overall specificity was lower since there were 1,000 additional “Decoy” isolates (Table 4 and Table 5). For the “Decoy” serotype, the sensitivity and specificity were 74.7% and 91.7%, respectively. The accuracies were 95.9% and 96.1% by excluding and including the “Decoy” isolates, in the calculation, respectively. The relationships among the 14 subgroups were further analyzed using the hierarchical cluster using the Euclidean distance function and the average agglomeration method (Figure 6). The 14 subgroups identified all 5 major serotypes and their subtypes, and the “Decoy” serotype: 1. Thompson (0010000000); 2. Typhimurium (0100000000); 3. Decoy (0000000000); 4. Oranienburg contained 5 subtypes (0001000000, 0000100000, 0001100000, 0001110000, 0000110000); 5. Hadar contained 2 subtypes (0000010000, 1000010000); 6. I4,[5],12:i- contained 4 subtypes (1000000000, 1000000100, 1000001000, 0000001000).

**Table 5:** Frequency distributions of subgroup patterns identified by the SVM composite model for the Salmonella PFGE test dataset, which consisted of 5,055 isolates from five training serotypes and 1,000 additional “Decoy” isolates.

14subgroups*(n≥5)*	An = 1156	Bn = 992	C*n = 930*	Dn = 1047	En = 930	Fn = 1000	Totaln = 6055
0000000000	38	73	122	56	133	747	1169
1000000000	699	0	*0*	0	1	176	876
1000000100	204	0	*0*	0	0	3	207
1000001000	204	0	*0*	0	0	11	215
0000001000	5	0	0	0	0	2	7
0100000000	0	0	0	0	795	3	798
0010000000	0	0	0	987	0	42	1029
0001000000	0	0	24	0	0	0	24
0000100000	0	0	216	0	0	1	217
0001100000	0	0	544	0	0	0	544
0001110000	0	0	8	0	0	0	8
0000110000	0	0	6	0	0	0	6
0000010000	0	911	9	1	0	10	931
1000010000	0	8	0	0	0	1	9
Minority (10)	6	0	1	3	1	4	15
Correct	1112	919	798	987	795	747	5358
Sensitivity	.962	.926	.858	.943	.855	.747	.885
Specificity	.961	.996	1.00	.992	.999	.917	.977

The serotypes I4,[5],12:i-, Hadar, Oranienburg, Thompson, Typhimurium, and Decoy were labeled as A, B, C, D, E, and F, respectively. n is the number of isolates in the serotypes. Fourteen of 24 identified classification patterns had frequencies at least 5. The last two rows show the sensitivity and specificity of the model performance.

The PFGE test data were further analyzed using the k-means clustering to identify serotypes and their subtypes, and the SVM and RF algorithms to predict serotypes (including 1,000 Decoy isolates). Table 6 shows the sensitivity, specificity and accuracy of the three procedures. The k-means analysis was performed for k = 5 to 15; only the results for k = 5, 6, 10, and 15 are presented. The k-means analysis was also based on majority rule to determine the serotype. The k-means’ performances were similar except for k = 5, in which the number of clusters were mis-specified. It appears that k-means has generally better performance than the composite model, except when a smaller k is specified. The SVM has much better performance than either the composite model or k-mean methods for the test dataset without Decoy data, the accuracy is more than 99%. The SVM is unable to predict the Decoy data since their serotypes are not in the training classes.

**Table 6:** Sensitivity (SN), specificity (SP), and accuracy of the composite model, k-means, and support vector machine (SVM) procedures for the PFGE test dataset.

		A	B	C	D	E	F	ACC	
SVM Composite	SN	.962	.926	.858	.943	.855	.747	.885	
	SP	.961	.996	1	.992	.999	.917		
RF	SN	.960	.927	.847	.945	.873	.750	.894	
Composite	SP	.961	.996	1	.992	.991	.921		
5-means	SN	.997	.976	.981	.973	.975	0	.819	
	SP	.919	.955	.960	.974	.974	1		
6-means	SN	.960	.947	.932	.966	.917	.816	.924	
	SP	.966	.996	.999	.999	.999	.947		
10-means	SN	.891	.941	.919	.976	.895	.941	.927	
	SP	.989	.999	.999	.999	1	.924		
15-means	SN	.900	.942	.916	.954	.952	.944	.934	
	SP	.990	.999	.999	.999	.999	.933		
SVM	SN	.999	.985	.998	.995	1	0	.831	
	SP	.945	.975	.970	.966	.942	1		
RF	SN	.999	.994	.998	.996	.999	0	.832	
	SP	.941	.967	.954	.963	.974	1		

Serotypes A–F are defined in Table 4.

Currently, PFGE is routinely used molecular subtyping method by CDC (Centers for Disease Control and Prevention) and state health labs in the US for *Salmonella* surveillance and outbreak investigation [52], the ability to rapidly identify the serotype or a subtype of a *Salmonella* isolate is essential. The same serotype may have different subtypes, such as *Salmonella* Newport, and Dublin etc. These subtypes are closely related with their gene composition and variations. Current routine serotyping methods cannot provide sufficient information for subtype classification. The serotype subtype classification is important for the studies of genetic diversity and evolution. The composite model not only contributes to the PFGE-based characterization and surveillance of *Salmonella* isolates in outbreak investigations, also provides a better understanding of *Salmonella* genetic diversity and epidemiology.

## Discussion

Cluster analysis has been the primary data mining technique for dividing samples into disjoint subgroups where the samples in a cluster contain all attributes that characterize the cluster. Bicluster analysis techniques are being developed to identify which subsets of attributes are associated with which subsets of samples [34]–[39]. A bicluster analysis divides the samples into disjoint subgroups, where each sample in the subgroup corresponds to one or more subsets of attributes; and where there may be one additional subgroup formed by the samples not in any biclusters which are not associated with any subset of attributes. Both cluster analysis and bicluster analysis are powerful techniques for classifying samples into subgroups, but they are inefficient for prediction purpose. Either method can predict new samples by pooling the current samples with new samples then performing the same analysis. However, the subgroup membership of a current sample before and after the pooling may be different. Alternatively, either method may also assign the new sample using a classification algorithm such as k-NN (k-Nearest Neighbors) to develop a prediction model; note that k-NN requires specification of k and a distance measure between the new samples and the subgroups.

In the analysis of the lung cancer and PFGE datasets, Tables 4 and 5 show that k-means can outperform the proposed procedure when the number of clusters are correctly specified; however, it is often difficult to determine k when the sample size or the number of subgroups is large such as the PFGE data. Clustering analysis does not perform well if there is a subgroup of samples that are made of diverse subtypes, e.g., Decoy subgroup. The major advantages of the proposed procedure over k-means are: 1) it does not require pre-specifying the number of clusters, and 2) it uses a subset of attributes for each bicluster, instead of entire set of attributes, to develop a binary classifier. The composite model further identifies the relationships among subgroups based on their patterns of partition. Figure 6 clearly shows six distinct classes representing five serotypes and their sub-serotypes, and an unknown serotypes group. Finally, the hierarchical clustering tree can provide relationships among the clusters by a cutoff however, there seems to have no standard criterion or algorithm for choosing a cutoff; the cutoff is often made by visual inspection. When the number of samples and/or the number of clusters is large, such as the PFGE data, the visual inspection becomes infeasible.

Biclustering algorithms have been extended to supervised biclustering classification for labelled sampled data [43]–[46]. There are the CCC-biclustering algorithm to classify good versus poor responders [43], the co-clustering algorithm to discriminate between two sample classes (Class A versus Class B) [44], the subspace co-expression analysis to discover differential co-expression patterns to classify normal versus cancer samples [45], and the LAS (large average submatrix) to classify five breast cancer subtypes [46]. These methods are supervised biclustering-based classifiers (or class-discriminant biclusters) [43], classification algorithms which were developed while optimizing the class discriminative ability from the label information. A two-class supervised biclustering algorithm can be extended to a multiclass classification algorithm. However, classification algorithms are unable to characterize the subgroup relationships without further analysis. The composite model considers unlabeled data; the objectives are not only to classify samples into subgroups and predict new samples but also to characterize the relationships among subgroups. Recently, Geraci et al. [53] proposed “Butterfly”, a discrete dynamic system, for visualization, clustering, and classification of unlabeled data. Butterfly provided a 2D representation of the relationship between samples according to a set of variables. The system first generated a set of 2D cluster models, after performing a feature reduction step, and evaluated by binary classifiers, and finally showed the visual representation of the top classification models On the other hand, the composite model is a general procedure applicable for two-class or multiclass prediction using biclusters with or without feature reduction.

In the proposed approach, a binary classifier is developed to predict whether or not a sample is in the associated bicluster. For the samples that are assigned into two or more biclusters, the composite model will separate those samples into a new subgroup. Some classifiers, either by itself or in combination with other classifiers, may assign only a small number samples, or none, into a subgroup. The PFGE analysis appeared to support some comments of Odibat and Reddy [44] that the biclustering approach itself is inadequate for subgroup discrimination. The Oranienburg serotype consisted of at least 5 subtypes (Table 5). It would need three biclusters, C_4_, C_5_, and C_6_, to identify (discriminate between) these subtypes. For example, the two biclusters C_5_ and C_6_ in combination identified seven Oranienburg isolates. In addition, the bicluster C_9_ and C_10_ were not shown in any of the 14 patterns.

The composite model uses k biclusters as a basis to generate up to 2^k^ disjoint subgroups. Those small biclusters are too small to be considered as representative subgroups for further partition. The composite model assigns each sample to one and only one subgroup, including those samples in the small biclusters. In the lung cancer example, the composite model was composed of three binary classifiers from three “large” biclusters of at least ten samples, out of the 32 biclusters identified. These three binary classifiers could generate up to 8 subgroups. However, only three subgroup patterns were identified. The smallest subgroup (0,1,0) contained only two samples (Table 2). Similarly, in the breast cancer example, two “large” biclusters were used. There were two small subgroups containing three and six samples. In the PFGE example, the composite model identified 16 subgroups based on 10 biclusters in the training dataset (Table 4). The numbers of the samples in the three smallest subgroups were 2, 2, and 3. The model identified 24 patterns in the training dataset (Table 5). The total number of samples for 10 smallest subgroups combined was 15, less than 2 on the average. The composite model is capable of identifying small subgroups.

Specification of the threshold n* can be based on the sample size and study objectives. For example, in personal medicine applications, patients are typically classified as high-risk versus low-risk or responders versus non-responders. The subgroups are identified for treatment recommendation. Different cancer subtypes or risk groups are subjected to different treatments. In the lung cancer example, the treatments for the two subtypes are different. In the breast cancer example, patients in the high risk group would be recommended to more aggressive treatment. In both examples, n* was set at ten. In the PFGE example, the primary objective was to develop a model to identify/predict serotypes/subtypes of unknown isolates. Knowing that there were many subtypes, n* was set at five. Ten biclusters were used to develop ten classifiers. The three small “subgroups” with sizes 2, 2, and 3 can be further investigated, if necessary.

In this paper, a minimum of five samples is recommended, n* = 5. In the lung cancer example, three biclusters with sample sizes of 40, 22, and 10 were used to generate subgroups. Cluster C_3_ consisted of 10 samples. As discussed, the prediction results by m_3_ were that all 10 samples were outside the C_3_ bicluster. These 10 samples were assigned primarily based on the classifiers m_1_ and m_2_. In other words, biclusters C_1_ and C_2_ were sufficient to develop the composite model in the sample assignment. In general, the samples from small biclusters are likely to be assigned to some larger biclusters. An explanation is that there are much more samples outside the bicluster region than the samples inside; a binary classifier tends to favor the majority class prediction in order to maximize total accuracy. Smaller biclusters (n<5) can be used to develop a composite model. However, classifier developed by a small bicluster is likely to predict that the samples are outside the bicluster. This problem is known as class-imbalanced classification [54]. Furthermore, for large binary data matrix, there may be hundreds of 2×2, 2×3, 3×2, and 3×3 biclusters.

The notion of the composite modeling approach via biclusters for class prediction is intuitive and straightforward. For a given bicluster, a sample is either inside or outside the bicluster. There are k predicted outcomes for each sample. Each predicted pattern represents a subgroup. In the simulation experiment, four biclusters C_1_–C_4_ were identified. The sizes of C_1_–C_4_ were 100×16, 50×51, 50×58, and 100×15, respectively. Samples 1–50 and samples 41–90 were in biclusters C_3_ and C_2_, respectively; and samples 41–50 appeared in all four biclusters C_1_–C_4_. Table 1 shows that the composite model performed well in classification of the sample 1–90 since the four binary classifiers were developed based on the four biclusters. Samples 91–100 were not in any of the four biclusters, these samples are not associated with any subsets of attributes. In the PFGE data, there were 10 biclusters with the sizes: 8×1097, 13×813, 9×938, 10×596, 5×787, 10×938, 5×175, 3×178, 3×468, and 2×109. There were many overlapping biclusters. These biclusters represented relative large numbers of samples with small numbers of attributes. On the other hand, in the lung data, the three biclusters with the sizes of 55×40, 18×22, and 4×10 were smaller biclusters relatively. In the simulation, lung cancer and PFGE examples, where the subgroups were known, the SVD-based biclustering algorithm was able to capture the critical subgroup structures. The composite model appeared to perform reasonable well. In the proposed approach, any types of bicluster patterns and any biclustering methods can be used to develop a composite model. However, the performance of a composite model highly depends on the biclusters used to generate binary classifiers. A good biclustering method is essential for the next step of subgroup classification and prediction.

The three classification algorithms, SVM, RF, and DLDA, are considered for the development of a composite prediction model. The SVM and RF have been the most popular and successful classification algorithms and applied to numerous areas of applications. These two algorithms can be applied to high dimensional data without feature selection. DLDA is a variant of the Fisher’s linear discriminant analysis. DLDA has been shown to be robust against imbalanced class size data [54], where the numbers of samples in the bicluster and outside differs substantially. When the class sizes are imbalanced, the standard classification algorithms, such as SVM and RF, will favor majority class prediction resulting in poor performance. Among the three algorithms, SVM appears to perform consistently well.

Personalized medicine is the goal of much current research. A general aim is to identify a set of molecular biomarkers that can match disease of an individual patient with an optimal therapy. Several procedures have been proposed utilizing the classification and regression tress [19] for subgroup identification. These procedures partitioned the entire covariate space into subsets of patients that are homogeneous with respect to the set of covariates [55]–[58]. This paper proposes a composite prediction model as an alternative procedure to classify samples into subgroups according their associated attributes. Unlike the supervised classification tree approach, the proposed procedure is an unsupervised approach. The procedure provides an approach to classifying patients into subgroups of having different outcomes of interest, such as genotypic factors, phenotypic outcomes, efficacy/safety measures, or responses to treatments; the relationships among the subgroups identified can be further examined [59], [60]. However, the approach presented does not consider outcome measures that are associated with specific drug treatment. In other words, the applications focus on the prognostic model, not predictive model, in the context of personalized medicine [48].

## Supporting Information

Figure S1 The prediction model divided the 97 patients into four subgroups using RF. The logrank test for differences among the four subgroups (0,0), (0,1), (1,0), and (1,1) was 0.717.
(TIF)

Figure S2 The prediction model divided the 97 patients into four subgroups using DLDA. The logrank test for differences among the four subgroups (0,0), (0,1), (1,0), and (1,1) was 0.186.
(TIF)

Table S1 Upper panel. Frequency distributions of classification patterns identified by the RF composite model (m1, m2, m3, m4) for the synthetic training dataset consisting of 4 subgroups, S1, S2, S3, and S4. Lower panel. Performance of the RF composite prediction model for the test dataset of 1,000 simulated samples. Table values are the averages over 1,000 repetitions.
(DOC)

Table S2 Upper panel. Frequency distributions of classification patterns identified by the DLDA composite model (m1, m2, m3, m4) for the synthetic training dataset consisting of 4 subgroups, S1, S2, S3, and S4. Lower panel. Performance of the DLDA composite prediction model for the test dataset of 1,000 simulated samples. Table values are the averages over 1,000 repetitions.
(DOC)

Table S3 Frequency distributions of classification patterns identified by the RF composite model (m_1_–m_10_) for the *Salmonella* PFGE training dataset consisting of five serotypes. Sixteen classification patterns are identified; 13 of the 16 have frequencies of at least 5 (last column). The last two rows show the sensitivity and specificity of the model performance.
(DOC)

Table S4 Frequency distributions of subgroup patterns identified by the RF composite model for the Salmonella PFGE test dataset, which consisted of 5,055 isolates from five training serotypes and 1,000 additional “Decoy” isolates. The serotypes I4,[5],12:i-, Hadar, Oranienburg, Thompson, Typhimurium, and Decoy were labeled as A, B, C, D, E, and F, respectively. n is the number of isolates in the serotypes. Fourteen of 24 identified classification patterns had frequencies at least 5. The last two rows show the sensitivity and specificity of the model performance.
(DOC)
