# Supplementary figures and images for: A Composite Model for Subgroup Identification and Prediction via Bicluster Analysis
Source: PLoS One. 2014 Oct 27;9(10):e111318. doi: 10.1371/journal.pone.0111318 (PMC4210136; doi:10.1371/journal.pone.0111318)

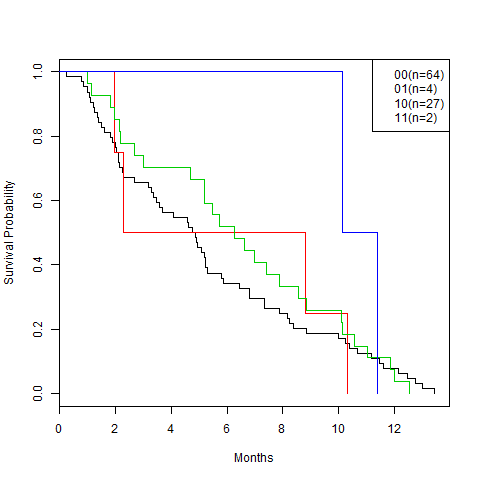

Supplement: Figure S1 — The prediction model divided the 97 patients into four subgroups using RF. The logrank test for differences among the four subgroups (0,0), (0,1), (1,0), and (1,1) was 0.717. (TIF) [file pone.0111318.s001.tif]

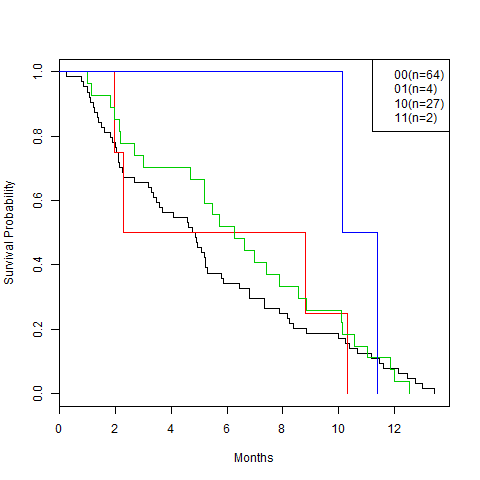

Supplement: Figure S2 — The prediction model divided the 97 patients into four subgroups using DLDA. The logrank test for differences among the four subgroups (0,0), (0,1), (1,0), and (1,1) was 0.186. (TIF) [file pone.0111318.s002.tif]
