# Supplementary material for: A Composite Model for Subgroup Identification and Prediction via Bicluster Analysis
Source: PLoS One. 2014 Oct 27;9(10):e111318. doi: 10.1371/journal.pone.0111318 (PMC4210136; doi:10.1371/journal.pone.0111318)
Supplement: Table S2 — Upper panel. Frequency distributions of classification patterns identified by the DLDA composite model (m1, m2, m3, m4) for the synthetic training dataset consisting of 4 subgroups, S1, S2, S3, and S4. Lower panel. Performance of the DLDA composite prediction model for the test dataset of 1,000 simulated samples. Table values are the averages over 1,000 repetitions. (DOC) [file pone.0111318.s004.doc]

**Table S2.**Upper panel. Frequency distributions of classification patterns identified by the DLDA composite model (m1, m2, m3, m4) for the synthetic training dataset consisting of 4 subgroups, S1, S2, S3, and S4. Lower panel. Performance of the DLDA composite prediction model for the test dataset of 1,000 simulated samples. Table values are the averages over 1,000 repetitions.

| Subgroup Pattern | S1  (n=40) | S2  (n=10) | S3  (n=40) | S4  (n=10) | Total  (n=100) |
| --- | --- | --- | --- | --- | --- |
| **Training** |  |  | | | |
| 0000 | 0 | 0 | 0 | 1 | 1 |
| 0001 | 0 | 0 | 0 | 2 | 2 |
| 0010 | 37 | 0 | 0 | 0 | 37 |
| 0011 | 3 | 0 | 0 | 0 | 3 |
| 0100 | 0 | 0 | 40 | 3 | 43 |
| 0101 | 0 | 0 | 0 | 3 | 3 |
| 0110 | 0 | 0 | 0 | 1 | 1 |
| 1111 | 0 | 10 | 0 | 0 | 10 |
| Sensitivity | 0.925 | 1 | 1 | 0 | 0.870 |
| Specificity | 1 | 1 | 0.95 | 1 | 0.990 |
| **Test** |  |  | | | |
| Sensitivity | 0.996 | 0.993 | 0.996 | 0.567 | 95.3 |
| Specificity | 0.967 | 0.996 | 0.967 | 0.996 | 98.5 |
