# Supplementary material for: A Composite Model for Subgroup Identification and Prediction via Bicluster Analysis
Source: PLoS One. 2014 Oct 27;9(10):e111318. doi: 10.1371/journal.pone.0111318 (PMC4210136; doi:10.1371/journal.pone.0111318)
Supplement: Table S3 — Frequency distributions of classification patterns identified by the RF composite model (m1–m10) for the Salmonella PFGE training dataset consisting of five serotypes. Sixteen classification patterns are identified; 13 of the 16 have frequencies of at least 5 (last column). The last two rows show the sensitivity and specificity of the model performance. (DOC) [file pone.0111318.s005.doc]

**Table S3.**  Frequency distributions of classification patterns identified by the RF composite model (m_1_ − m_10_) for the *Salmonella* PFGE training dataset consisting of five serotypes. Sixteen classification patterns are identified; 13 of the 16 have frequencies of at least 5 (last column). The last two rows show the sensitivity and specificity of the model performance.

| **12 subgroups**  (**n ≥ 5)** | **4,5,12:i-n=1113** | **Hadar**  **n=982** | **Oranienburg**  **n=997** | **Thompson**  **n=990** | **Typhi**  **n=972** | **Total**  **n=5054** |
| --- | --- | --- | --- | --- | --- | --- |
| 0000000000 | 38 | 71 | 130 | 47 | 134 | **420** |
| 1000000000 | 898 | 0 | 0 | 0 | 0 | **898** |
| 1000001000 | 166 | 0 | 0 | 0 | 0 | **166** |
| 0000001000 | 8 | 0 | 0 | 0 | 0 | **8** |
| 0100000000 | 0 | 0 | 0 | 0 | 836 | **836** |
| 0010000000 | 0 | 0 | 0 | 942 | 0 | **942** |
| 0001000000 | 0 | 0 | 34 | 0 | 0 | **34** |
| 0000100000 | 0 | 0 | 195 | 0 | 0 | **195** |
| 0001100000 | 0 | 0 | 612 | 0 | 0 | **612** |
| 0001110000 | 0 | 0 | 9 | 0 | 0 | **9** |
| 0000110000 | 0 | 0 | 9 | 0 | 0 | **9** |
| 0000010000 | 1 | 908 | 8 | 1 | 2 | **919** |
| 1010000000 | 1 | 0 | 0 | 0 | 0 | **1** |
| 0000011000 | 1 | 0 | 0 | 0 | 0 | **1** |
| 0010010000 | 0 | 3 | 0 | 0 | 0 | **3** |
| **Correct identification** | **1110** | **908** | **859** | **942** | **970** | **4751** |
| **Sensitivity** | **0.997** | **0.925** | **0.862** | **0.952** | **0.998** | **0.940** |
| **Specificity** | **1** | **0.997** | **1** | **1.000** | **0.930** | **0.982** |
