# Supplementary material for: A Composite Model for Subgroup Identification and Prediction via Bicluster Analysis
Source: PLoS One. 2014 Oct 27;9(10):e111318. doi: 10.1371/journal.pone.0111318 (PMC4210136; doi:10.1371/journal.pone.0111318)
Supplement: Table S4 — Frequency distributions of subgroup patterns identified by the RF composite model for the Salmonella PFGE test dataset, which consisted of 5,055 isolates from five training serotypes and 1,000 additional “Decoy” isolates. The serotypes I4,[5],12:i-, Hadar, Oranienburg, Thompson, Typhimurium, and Decoy were labeled as A, B, C, D, E, and F, respectively. n is the number of isolates in the serotypes. Fourteen of 24 identified classification patterns had frequencies at least 5. The last two rows show the sensitivity and specificity of the model performance. (DOC) [file pone.0111318.s006.doc]

**able S4.** Frequency distributions of subgroup patterns identified by the RF composite model for the Salmonella PFGE test dataset, which consisted of 5,055 isolates from five training serotypes and 1,000 additional “Decoy” isolates. The serotypes I4,[5],12:i-, Hadar, Oranienburg, Thompson, Typhimurium, and Decoy were labeled as A, B, C, D, E, and F, respectively. n is the number of isolates in the serotypes. Fourteen of 24 identified classification patterns had frequencies at least 5. The last two rows show the sensitivity and specificity of the model performance.

| **13 subgroups**  ***(n ≥ 5)*** | **A**  **n=1156** | **B**  **n=992** | **C**  **n=930** | **D**  **n=1047** | **E**  **n=930** | **F**  **n=1000** | **Total**  **n=6055** |
| --- | --- | --- | --- | --- | --- | --- | --- |
| **0000000000** | 43 | 65 | 121 | 54 | 116 | 750 | **1149** |
| **1000000000** | 901 | 0 | 0 | 0 | 1 | 177 | **1079** |
| **1000001000** | 204 | 0 | 0 | 0 | 0 | 11 | **215** |
| **0000001000** | 5 | 0 | 0 | 0 | 0 | 2 | **7** |
| **0100000000** | 0 | 0 | 0 | 0 | 812 | 3 | **815** |
| **0010000000** | 0 | 0 | 0 | 989 | 0 | 44 | **1033** |
| **0001000000** | 0 | 0 | 25 | 0 | 0 | 0 | **25** |
| **0000100000** | 0 | 0 | 193 | 0 | 0 | 1 | **194** |
| **0001100000** | 0 | 0 | 570 | 0 | 0 | 0 | **570** |
| **0001110000** | 0 | 0 | 6 | 0 | 0 | 0 | **6** |
| **0000110000** | 0 | 0 | 6 | 0 | 0 | 0 | **6** |
| **0000010000** | 0 | 920 | 9 | 1 | 0 | 10 | **940** |
| **1000010000** | 0 | 7 | 0 | 0 | 0 | 0 | **7** |
| **Minority (7)** | **3** | **0** | **0** | **3** | **1** | **2** | **9** |
| **Correct** | **1110** | **920** | **788** | **989** | **812** | **750** | **5412** |
| **Sensitivity** | **.960** | **.927** | **.847** | **.945** | **.873** | **.750** | **.894** |
| **Specificity** | **.961** | **.996** | **1.00** | **.992** | **.991** | **.921** | **.978** |
